# Astrocyte heterogeneity in brain metastases

**DOI:** 10.1002/1878-0261.70296

**Published:** 2026-07-08

**Authors:** Carolina Hernández‐Oliver, Neibla Priego, Manuel Valiente

**Affiliations:** ^1^ Brain Metastasis Group Spanish National Cancer Research Centre (CNIO) Madrid Spain

**Keywords:** astrocytes, brain metastasis, cellular states, heterogeneity, microenvironment, therapeutic targets

## Abstract

Brain metastasis is increasingly understood as a disease of reciprocal adaptation between metastatic cells and the brain microenvironment. Among host cells, astrocytes have emerged as central regulators of metastatic colonization, survival, immune remodeling, and treatment resistance. Recent studies are re‐defining astrocytes to explore the functional implications of their cellular heterogeneity. Evidence in brain metastasis indicates that astrocyte responses are heterogeneous across anatomical space, stage of colonization, and tumor type. This framework helps explaining how distinct astrocyte programs can coexist and why no single experimental platform captures their organization and complex biology. Moving toward spatially resolved, longitudinal, and functionally validated definitions of astrocyte states could clarify mechanisms and guide more precise therapeutic opportunities in brain metastasis. In this Viewpoint, we examine studies that have challenged the long‐standing paradigm of astrocytes as a uniform entity, highlighting how recent technological advances reinforce previous findings and pave the way for identifying new therapeutic targets.

## Graphical abstract

Astrocytes emerge as pivotal regulators of metastatic colonization, survival, immune remodeling, and therapy response associated with an increasing heterogeneity that requires spatially and longitudinally resolved approaches to uncover regulatory programs and guide context‐specific therapies.

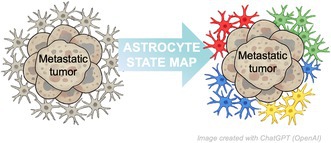

**Abbreviations**
AMP: Adenosine Monophosphate
CCL2: C‐C Motif Chemokine Ligand 2
Cdk5: Cyclin‐Dependent Kinase 5
cGAMP: Cyclic GMP‐AMP
CNS: Central Nervous System
EGFR: Epidermal Growth Factor Receptor
GMP: Guanosine Monophosphate
GPX3: Glutathione Peroxidase 3
IL‐1β: Interleukin‐1 Beta
LPS: Lipopolysaccharide
mGluR1: Metabotropic Glutamate Receptor 1
MHC‐I: Major Histocompatibility Complex Class I
miR‐19a: microRNA‐19a
PDGFRβ: Platelet‐Derived Growth Factor Receptor Beta
PTEN: Phosphatase and Tensin Homolog
SCLC: Small Cell Lung Cancer
scRNA‐seq: Single‐Cell RNA Sequencing
snRNA‐seq: Single‐Nucleus RNA Sequencing
STAT3: Signal Transducer and Activator of Transcription 3
STING: Stimulator of Interferon Genes
Th17: T Helper 17
Wnt5a: Wnt Family Member 5A

## Introduction

### Astrocyte heterogeneity: One key missing framework in brain metastasis

For many years, brain metastasis research focused primarily on the genomic evolution and phenotypic plasticity of metastatic cancer cells [1, 2, 3, 4]. It is now equally clear, however, that successful colonization of the brain also depends on active cooperation from the host microenvironment, with astrocytes emerging as central regulators of metastatic fitness [4, 5, 6]. Foundational studies have already demonstrated that astrocytes are not passive bystanders in this process. Astrocytes are involved in blood–brain barrier remodeling and vascular co‐option [7, 8], can control brain invasion [9, 10, 11, 12], boost brain metastasis metabolism [13], support metastatic survival, outgrowth and chemoresistance through a variety of mechanisms including exosome‐mediated PTEN suppression in tumor cells, paracrine signaling and gap‐junction communication as well as local immunomodulation through STAT3‐dependent and independent mechanisms [14, 15, 16, 17, 18, 19, 20, 21]. Taken together, these observations have largely resolved one question for the field: the issue is no longer whether astrocytes matter in brain metastasis, but how their contributions are organized at the functional level.

Importantly, the mechanistic diversity already reported argues against treating astrocytes as a single functional entity. Robust evidence for the coexistence of distinct molecular programs in astrocytes challenges the notion that all astrocytes perform the same role across all lesions, anatomical regions, and stages of metastatic progression. While a comprehensive map is still lacking, the observed heterogeneity of astrocyte–tumor interactions strongly indicates functional non‐equivalence among astrocytes in the metastatic brain microenvironment [15, 16, 19, 20, 21, 22, 23, 24]. In this sense, the persistent use of the umbrella term *reactive astrocytes* risks obscuring the very biology that now seems most important.

The broader astrocyte field at present provides the conceptual framework needed to address this gap. Work in healthy brain tissue has shown that astrocytes are intrinsically regionally diverse, with distinct transcriptional programs distributed across anatomical territories [25, 26]. In parallel, studies across CNS disorders have made clear that astrocyte reactivity is not a single stereotyped phenotype, but a context, space, and time‐dependent spectrum of states. This view is supported by mechanistic and single‐cell work largely conducted in mouse systems [27], alongside community efforts to standardize definitions and nomenclature across contexts and species [28], and is reinforced by recent reviews synthesizing evidence for multi‐state reactivity [29, 30].

Brain metastasis research, therefore, stands at a conceptual turning point: rather than interpreting astrocyte involvement through a unitary “reactive astrocyte” label, it should be understood through an astrocyte state map, a framework that considers which astrocyte states arise, where they are positioned, when they emerge, and how they influence distinct steps of metastatic colonization and progression. In this view, heterogeneity is not a side note in brain metastasis biology, but the missing bridge between established astrocyte involvement and unresolved mechanism. When moving from an uninjured brain to the context of brain tumors, and particularly brain metastases, it is important to consider the availability of models that faithfully recapitulate the disease, as well as the accessibility and processing of human samples. These factors will be decisive for advancing the study of the diverse aspects in astrocyte functional heterogeneity.

To enable a consistent interpretation of functional heterogeneity in this context, the following key concepts are used throughout this review: state refers to a context‐dependent transcriptional program observed *in situ*, which may be continuous or trajectory‐like, and program denotes the underlying gene module or regulatory axis. In contrast, subpopulation is used when a given state reproducibly maps to a spatially restricted and/or functionally definable subset of cells.

### Reactive astrocytes encode multiple functional programs in brain metastasis

Several pivotal studies already support the idea that astrocyte responses in brain metastasis are functionally dynamic. Even before the field had adopted a formal heterogeneity framework, foundational work was already describing distinct astrocyte‐dependent mechanisms that are difficult to reconcile with a single, uniform reactive entity. Zhang et al. showed that astrocytes can promote metastatic outgrowth by transferring exosomal miR‐19a to tumor cells, thereby inducing reversible PTEN downregulation, increasing CCL2 production, and enhancing myeloid cell recruitment in the metastatic niche [15]. Chen et al. identified a different mode of astrocyte support, in which breast and lung cancer cells establish protocadherin‐7‐ and connexin‐43‐dependent gap junctions with astrocytes, enabling cGAMP transfer, STING activation, and downstream IFN‐α and TNF‐α signaling that enhance tumor survival and chemoresistance [16]. Together, these studies established astrocytes as active effectors of metastatic fitness, but they also implied something more fundamental: astrocyte involvement in brain metastasis is mechanistically diverse from the outset, and therefore unlikely to be explained by a single reactive program.

Other studies represented a conceptual shift in the field, refining the view of astrocyte involvement in metastasis from a generalized supportive role to the notion that discrete astrocyte states contribute to metastatic progression. In this context, STAT3 emerged not merely as another signaling pathway, but as a defining feature of a specific reactive astrocyte compartment linked to a pro‐metastatic function. Priego et al. showed that a subset of reactive astrocytes surrounding metastatic lesions displays activated STAT3 and is required for metastatic progression. These astrocytes promote tumor growth by shaping both innate and adaptive immune responses, and their presence correlates with shorter survival after diagnosis of intracranial metastasis in patients [19, 24, 31]. This study provides direct evidence linking pro‐metastatic astrocyte activity to a defined reactive compartment. Consistent with this, prior work identified a perivascular subpopulation of metastasis‐associated astrocytes defined by p751‐PDGFRβ, suggesting a targetable astrocyte state involved in early metastatic outgrowth [32].

More recent studies suggest that the astrocyte response in brain metastasis extends beyond the STAT3‐positive compartment, although these additional programs have not yet been positioned relative to one another in a unified framework. Ma et al. reported that cancer‐activated astrocytes generate a sustained low‐level type I interferon niche that enhances CCL2‐dependent recruitment of monocytic myeloid cells and promotes brain metastasis in breast cancer and melanoma models [20]. In small‐cell lung cancer brain metastasis, Qu et al. showed that tumor–astrocyte crosstalk partially recapitulates developmental programs: SCLC cells recruit astrocytes through Reelin, and astrocytes in turn support tumor survival through factors such as SERPINE1 [33]. Ishibashi et al. further demonstrated that astrocyte‐derived Wnt5a induces mGluR1 expression in lung cancer brain metastasis cells, creating a glutamate‐dependent mechanism that stabilizes EGFR and supports metastatic growth [34]. In breast cancer brain metastasis, Yuzhalin *et al*. described an astrocyte‐driven immune‐evasion circuit in which astrocyte‐derived signals induce Cdk5 in tumor cells, suppressing MHC‐I expression and limiting immune recognition; notably, this study defines an additional astrocyte‐dependent regulatory axis, but not yet a clean astrocyte state marker *per se* [21]. Emerging work has also identified GPX3‐positive astrocytes in breast cancer brain metastasis, activated by circulating tumor cell‐derived exosomes and linked to IL‐1β production, Th17 differentiation and niche formation, providing an additional candidate astrocyte population that may prove relevant as the field matures [22].

Taken together, these studies indicate that reactive astrocytes in brain metastasis are not a single entity. They validate multiple astrocyte‐dependent pathways; however, functionally distinct astrocyte programs have yet to be fully integrated into a shared astrocyte state map. It remains unresolved whether the programs described are mutually exclusive, partially overlapping, sequentially deployed, lineage‐biased, tumor‐type‐specific, or stage‐specific. Addressing this gap will likely be important for understanding which astrocyte states are causal, when they arise, where they localize, and which of them may be therapeutically targetable.

### Heterogeneity across space, time, and tumor type

**Fig. 1:**
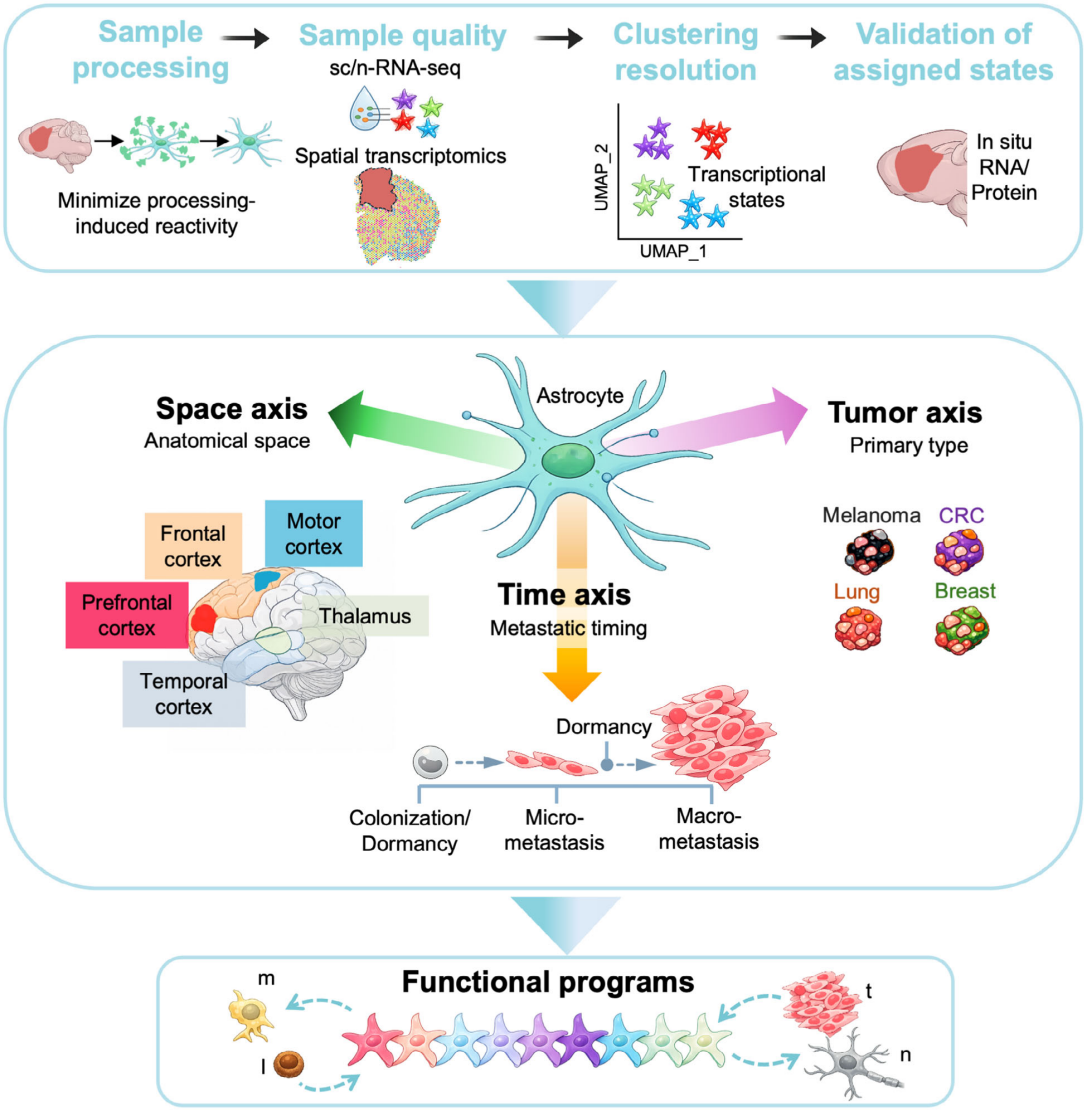
Integrating heterogeneity to define the astrocyte state map of brain metastasis. Upper panel summarizes key experimental and analytical challenges for defining astrocyte states in brain metastasis, from tissue processing to state validation. Tissue dissociation can induce *ex vivo* stress signatures, including aberrant “reactive” gene expression, which may confound downstream state inference. Astrocytes are frequently under‐recovered in dissociation‐based datasets relative to their abundance *in situ*, contributing to sampling bias and limiting power to detect rare or disease‐associated substates. Current profiling strategies include scRNA‐seq, snRNA‐seq, and spatial transcriptomics, the latter enabling anatomical localization of transcriptional programs within intact tissue. However, clustering outcomes remain sensitive to preprocessing and resolution choices, and the field lacks consensus on when clusters represent discrete states versus continuous variation. Intermediate panel depicts how astrocyte programs are context‐dependent across anatomical space, stage of metastatic colonization, and tumor type; robust state definition requires orthogonal validation. Recommended validation includes *in situ* RNA/protein confirmation (multiplex IF/ISH), spatial niche assignment (edge/core/perivascular), and functional perturbation to establish causality. Lower panel illustrates how heterogeneity across the three principal axes (space, time, and tumor type) gives rise to multiple functional programs and shapes interactions with other cell types within the metastatic niche (m: macrophages/microglia, l: leukocytes, n: neurons, and t: tumor), particularly through astrocyte‐driven immune remodeling. Astrocytes engage myeloid‐lineage cells (microglia and recruited peripheral myeloid cells) through multiple communication routes, including cytokine/chemokine signaling, exosome‐mediated transfer, and gap‐junction–dependent coupling. These circuits can reshape the local immune niche and influence metastatic fitness, consistent with reported STAT3‐driven immunosuppressive programs [19], cGAMP transfer through astrocyte–tumor gap junctions [16], and inflammatory recruitment axes [20]. AI (ChatGPT, OpenAI) generated image manually improved.

If reactive astrocytes are not one entity, the next question is what organizes their diversity in brain metastasis. A useful framework is to consider astrocyte heterogeneity along at least three axes: space, time, and tumor type (Fig. 1). Accordingly, this framework considers local brain architecture, disease progression, and tumor identity in determining which astrocyte states emerge and become functionally relevant [5, 6].

A first major axis is space. Work in the broader astrocyte field has shown that astrocytes are not interchangeable across the brain but are intrinsically regionally diverse. This has been demonstrated at high resolution in mouse models [25] and reinforced by large‐scale human brain atlases [26], with recent synthesis emphasizing how regional identity constrains astrocyte responses across context [35]. This principle is highly relevant to brain metastasis. Metastatic lesions arise in non‐equivalent anatomical contexts, including gray versus white matter regions, cortical versus subcortical territories, and vascular versus parenchymal niches. Because astrocytes differ across these territories, the local astrocyte repertoire available to a metastatic clone is unlikely to be uniform. Regional identity may accordingly shape which programs are preferentially engaged in a given lesion [36], whereas metastatic injury cues drive partial convergence toward shared reactive modules, a concept supported by clonal lineage work showing that astrocyte developmental history can yield divergent responses under a common insult [37]. In this context, it is critical to use brain metastasis models that preserve the spatiotemporal course of lesion initiation and outgrowth.

A second major axis is time. Astrocytes are unlikely to exert the same functions during seeding, dormancy, micrometastatic persistence and invasion, and macrometastasis outgrowth. This may help reconcile why astrocytes are sometimes described as restrictive [7, 38, 39] and at other times as overtly tumor‐supportive [15, 16, 19]: these observations may reflect different phases of the metastatic cascade rather than true biological contradiction. Notably, recent work suggests that temporal differences may be coupled to early spatial organization at outset, implying that astrocyte engagement may diverge already at early stages in a tumor subtype‐dependent manner [40]. Ma et al. showed that cancer‐activated astrocytes generate a sustained low‐level type I interferon niche during brain metastatic progression, promoting CCL2‐dependent recruitment of monocytic myeloid cells, while recent reviews emphasize that astrocyte functions evolve over the course of lesion establishment and progression [5, 6, 20]. Together, these findings suggest that astrocyte states should be understood as temporally staged and potentially dynamic, rather than as fixed responses.

A third major axis is tumor type. Most mechanistic work to date has focused on breast and lung cancer models, yet the astrocyte states engaged by HER2‐positive breast cancer, triple‐negative breast cancer, EGFR‐mutant lung adenocarcinoma, melanoma, and small‐cell lung cancer are unlikely to be interchangeable. The study by Qu et al. illustrates this especially well: in small‐cell lung cancer brain metastasis, tumor–astrocyte crosstalk partly recapitulates developmental programs, with SCLC cells using Reelin to recruit astrocytes and astrocytes subsequently secreting SERPINE1 to promote tumor survival [33]. Likewise, Ishibashi et al. showed that lung cancer brain metastases can exploit an astrocyte‐derived Wnt5a‐mGluR1 axis to stabilize EGFR and support metastatic growth, reinforcing the idea that astrocyte programs are, at least in part, tumor type‐conditioned [34]. Other studies have reported specific subpopulations independent of the primary tumor of origin, although the proportions of astrocytes within each program may vary between tumor types, with corresponding functional implications [19, 31]. This suggests that astrocyte heterogeneity in brain metastasis is not purely host‐intrinsic but emerges through bidirectional instruction between metastatic lineage and the organ‐specific niche. Within this perspective, it is highly relevant to investigate in depth brain metastases of rising incidence.

Taken together, these three dimensions argue that astrocyte heterogeneity in brain metastasis is best understood not as a static catalog of reactive programs, but as a context‐dependent organization shaped by anatomical position, metastatic timing, and tumor‐specific cues (middle panel Fig. 1). This is precisely why an astrocyte state map may be useful: it provides a framework for integrating mechanisms that might otherwise appear disconnected, and for asking whether distinct astrocyte programs are spatially restricted, temporally staged, lineage‐biased, or shared across metastatic settings. Without such a framework, astrocyte heterogeneity remains visible, but not yet fully interpretable [5, 6, 35].

### Experimental and analytical constraints in defining astrocyte states

Recent advances in single‐cell, single‐nucleus, and spatial transcriptomics have made astrocyte heterogeneity experimentally accessible and have been central to the shift from a generic “reactive astrocyte” concept toward a more resolved view of astrocyte states [27, 30, 35]. Most current insights come from standard scRNA‐seq and snRNA‐seq platforms, particularly 10 × Genomics and related droplet‐based methods, used in both targeted and untargeted workflows to improve astrocyte capture and increase the power to detect meaningful transcriptional differences, including rare subpopulations [12, 41, 42, 43]. More recently, sequencing‐based and imaging‐based spatial transcriptomic approaches have begun to restore anatomical context, allowing astrocyte states to be interpreted relative to brain region, lesion architecture, and local cellular neighborhoods [27, 44]. Large atlas studies further underscore this point: Siletti et al. profiled more than 150 000 astrocytes across multiple human brain regions and identified broad regional astrocyte classes directly linked to their anatomical location, reinforcing the idea that astrocyte identity is shaped by regional context even before disease occurs [26].

At the same time, these technologies impose important interpretive constraints (upper panel Fig. 1). Astrocytes are often underrepresented in dissociation‐based datasets relative to their abundance *in situ*, likely because their elaborate morphology and fine processes make them difficult to isolate and partition efficiently for single‐cell assays. Notably, neurons are broadly represented in scRNA‐seq datasets, so the precise basis of astrocyte under‐capture remains unsolved. Although single‐nucleus approaches may improve astrocyte recovery compared with whole‐cell dissociation in some settings [45], untargeted datasets still frequently contain relatively low astrocyte numbers, reducing the power to detect rare or disease‐associated populations and making results sensitive to enrichment strategy and dataset composition [46]. More fundamentally, tissue processing itself can distort inferred glial states. Marsh et al. showed that standard dissociation protocols can induce aberrant *ex vivo* glial signatures, underscoring how easily tissue handling can confound downstream analyses and inflate apparent cellular diversity [47]. On the other hand, strategies to enrich samples for astrocytes while preserving their integrity as much as possible, based on surface marker‐based separation, although widely accepted and used in the field [48], may alter the proportions of subpopulations, limiting the ability to fully recapitulate heterogeneity. In parallel, clustering‐based analyses remain highly dependent on preprocessing, integration, and resolution parameters, making it difficult to determine when a transcriptional cluster represents a robust biological state, a transient substate, or simply an analytic partition of continuous variation [28, 35]. In this context, orthogonal *in situ* validation at minimum protein‐level confirmation with spatial context remains indispensable. From a practical perspective, “astrocyte states” should be regarded as provisional entities until their combinatorial marker profiles are validated in intact tissue and consistently map onto well‐defined metastatic niches (upper panel Fig. 1).

Temporal resolution presents a further challenge. Studies in inflammatory and demyelinating settings have shown that astrocyte reactivity is dynamic, with gene expression programs that vary substantially across time and lesion context, suggesting that a state captured at one time point may reflect only one phase of a longer trajectory rather than a stable endpoint [27]. In the acute LPS model, for example, astrocytes remain transcriptionally altered for days after the initial insult, yet the specific genes that are differentially expressed change markedly across time points, arguing against the idea of a single fixed reactive program [27]. By analogy, brain metastasis studies that profile only established lesions cannot yet distinguish whether metastatic astrocytes largely retain their baseline homeostatic identity with selective transcriptional deviations, undergo progressive reprogramming as lesions evolve, or transition through temporally ordered states during seeding, persistence, and outgrowth. The goal, therefore, should not be to accumulate more astrocyte clusters, but to identify reproducible astrocyte states that are spatially localized, temporally interpretable, and functionally validated.

### Potential functional implications

Recognizing astrocyte heterogeneity is not merely descriptive; it shifts how we interpret astrocyte contributions in brain metastasis. Pro‐metastatic function can no longer be assumed to arise from all reactive astrocytes, but for specific astrocyte states which operate influenced by defined communication circuits (lower panel Fig. 1). Intercellular crosstalk is both a driver and a consequence of cellular heterogeneity, operating within a bidirectional framework in which communication circuits generate and are constrained by heterogeneous cellular states and their associated functional programs. For that, the key question becomes which astrocyte states are causally involved, when they emerge, and through which communication circuits they support metastatic progression [16, 19, 20, 24, 34]. Crucially, these circuits should be viewed as multicellular modules: *in vivo*, astrocyte programs are co‐determined by the reorganized cellular neighborhood, including microglia/TAMs, immune infiltrates, and neurons (5, 6). Microglia and monocyte‐derived macrophages display substantial heterogeneity in brain metastasis and could plausibly co‐pattern astrocyte states with specific myeloid programs, rather than generating a single shared reactive output [5, 49]. CD8+ T‐cell dysfunction and spatial restriction are increasingly documented in human brain metastases [50], and astrocyte‐defined immunosuppressive circuits provide direct mechanistic links between astrocyte state and local T‐cell function, such as the TIMP1 axis acting on infiltrating CD8+ T cells [24]. Finally, the expanding cancer neuroscience framework argues that metastatic lesions remodel local neural architecture and activity, which is likely to alter tonic neuron‐astrocyte signaling and metabolic coupling; this raises the possibility that neuronal context constrains or enables astrocyte state transitions in a niche‐dependent manner [51].

This distinction has immediate therapeutic implications. Broad anti‐astrocytic strategies are unlikely to be either effective or safe, because astrocytes are essential for neurotransmitter homeostasis, metabolic support, synaptic regulation, and vascular integrity in the normal brain. A more realistic goal is therefore not pan‐astrocyte suppression, but selective disruption of metastasis‐licensed astrocyte states or of the signaling circuits that sustain them. Several lines of evidence support the feasibility of selectively targeting defined astrocyte subpopulations. For instance, STAT3‐positive astrocytes have been shown to mediate local immunosuppression, and their inhibition disrupts pro‐metastatic immune remodeling [19], which is now being tested clinically (NCT05689619). Another astrocyte‐based clinical trial in brain metastases (NCT02429570) focused on targeting gap junction–mediated communication between astrocytes and tumor cells [16], as its disruption impairs the transfer of pro‐survival signals. In parallel, a perivascular subpopulation of PDGFRβ‐expressing astrocytes can be selectively reduced by pazopanib [32], pointing to a targetable state associated with early metastatic outgrowth. Additional pathways include endothelin receptor signaling, which contributes to astrocyte‐mediated chemoprotection of tumor cells [52], and Notch signaling, where pharmacological inhibition with Compound E limits astrocyte‐driven support of brain metastatic proliferation [18].

This framework may also help explain therapy resistance in brain metastasis. Astrocytes have already been implicated in protection from chemotherapy and in sustaining survival signaling in metastatic cells [52], but these effects may be better understood as ecosystem properties rather than generic consequences of astrocyte presence. If resistance is mediated by selected astrocyte states, transient support programs, or tumor type‐specific tumor–astrocyte interactions, then treatment failure should not be interpreted solely as a tumor‐cell‐autonomous phenomenon. Instead, it may reflect a cooperative niche in which metastatic cells exploit local astrocyte programs to buffer stress, evade immune pressure, or maintain growth signaling. This perspective supports combination strategies in which tumor‐directed therapies (or microenvironment‐directed therapies for the immune response) are paired with interventions against astrocyte‐dependent support circuits [24].

There is a compelling opportunity to leverage astrocyte heterogeneity for biomarker‐driven personalized medicine. Identification of secreted factors from specific pro‐metastatic astrocyte subpopulations could provide candidate biomarkers of response, while simultaneously revealing subpopulations that might be selectively targeted [24].

Ultimately, an astrocyte state framework shifts the field beyond the oversimplified notion of targeting all astrocytes toward the more precise goal of identifying reproducible, spatially localized, and functionally validated astrocyte vulnerabilities.

## Conclusions

Astrocyte heterogeneity is emerging as a central organizing principle in brain metastasis biology. Rather than viewing reactive astrocytes as a uniform response, the field should now move toward a state‐based framework that integrates transcriptional identity, spatial localization, temporal dynamics, intercellular communication, and functional relevance. This astrocyte state framework shifts the focus from targeting all astrocytes to identifying well‐defined and functionally validated astrocyte vulnerabilities.

In this regard, we consider the following priorities essential for the next stage of brain metastasis research.

First, refinement of brain metastasis research should transcend the traditional view of reactive astrocytes as GFAP‐positive cells and aim to define astrocytes within a structured, functionally informed state space. This will require integrated single‐cell, single‐nucleus, and spatial transcriptomic analyses in human specimens and clinically relevant models, ideally linked to perturbation experiments that test function rather than association alone [6, 30]. In practice, this argues for co‐profiling astrocytes and immune/myeloid compartments in the same spatial datasets, so that astrocyte state maps can be interpreted alongside microglia/TAM and T‐cell programs rather than in isolation. In parallel, the field should prioritize state‐linked perturbation (genetic or pharmacological) paired with spatial readouts, moving from correlative atlases toward causal circuit testing.

A second priority is annotation discipline. State labels borrowed from injury, neuroinflammation, or neurodegeneration may be informative, but they should not be applied uncritically to metastasis, where foreign‐cell recognition, vascular remodeling, corticosteroid exposure, radiotherapy, and systemic cancer signals create a distinct niche. Notably, insights from other CNS disorders are limited as metastasis may engage only a subset of those programs with additional tumor‐driven features that are not captured by existing nomenclature. Recent astrocyte reviews explicitly caution that transcriptomic diversity does not automatically define stable subtypes and that reactive states may be better understood as context‐dependent programs or trajectories rather than fixed categories [28, 29, 30].

A third priority is functional validation across progression. Current datasets still provide mostly snapshots, making it difficult to determine whether a reported astrocyte program reflects a persistent metastasis‐licensed state, a transient adaptation, or one phase of a longer trajectory. For brain metastasis, a useful next‐generation framework would therefore annotate astrocyte states not only by transcriptional profile but also by spatial localization, signaling behavior, temporal position, and impact on tumor fitness. That kind of framework would make it possible to compare lesions, lineages, and models on common terms rather than through loosely overlapping descriptions of “reactive astrocytes” [29, 30].

The ultimate goal of this research agenda is its translational impact: if only selected astrocyte states sustain metastatic growth or therapy resistance, the most promising therapeutic strategies are unlikely to be pan‐astrocytic. Instead, the field should prioritize metastasis‐relevant circuits that are both state‐enriched and pharmacologically tractable, including gap‐junction signaling, STAT3‐dependent programs, inflammatory myeloid‐recruiting axes, and lineage‐specific trophic interactions. A metastasis‐specific astrocyte state framework would help distinguish broadly reactive features from selective vulnerabilities and should therefore strengthen both mechanistic interpretation and therapy development [5, 6].

## Conflict of interest

M.V. receives research funds from AstraZeneca. The rest of co‐authors declare no conflicts of interest.

## Author contributions

CHO conceptualized the work and wrote the manuscript, MV and NP conceptualized the work, supervised, wrote, and revised the manuscript.
